# Supplementary material for: Patterns of Intron Gain and Loss in Fungi
Source: PLoS Biol. 2004 Nov 30;2(12):e422. doi: 10.1371/journal.pbio.0020422 (PMC532390; doi:10.1371/journal.pbio.0020422)
Supplement: Table S1 — Also available at http://genes.mit.edu/NielsenEtAl/. (4.3 MB ZIP). [file pbio.0020422.st001.zip › NielsenEtAl/html/295.html]

AN2999.1.NCU03857.1.MG07268.1.FG10347.1


```
 CLUSTAL W (1.82) Multiple Sequence Alignments - Introns Inserted


Sequence 1: NCU03857.1	462 aa
Sequence 2: MG07268.1	450 aa
Sequence 3: FG10347.1	445 aa
Sequence 4: AN2999.1	491 aa
Alignment Length: 495 aa
Number Identitical Residues: 314 aa
Alignment Score (without introns) 13213


MG07268.1 	------------------MRPAAS----------RVFGQSAFLARQ-SLLRPQQQQQLYA
NCU03857.1	------------------MSPVTANLIRSSLRQASVYSRLNIATAP-AFTRPAAPSLFVS
FG10347.1 	------------------MRRVAY----------------TSTTIR-PVRASFGAIQTIS
AN2999.1  	MSSVRFTSALARRSLNSPLLPCGAPPISSALRSFSSASYSAFSSSSSSFPSPSSARALAS
          	 ::   ::: :  : .:.:         ::  .    .     :  :..  .       :

MG07268.1 	AQ--------------FMRTMASQA--KIKVKNPVVELDGDE~MTRIIWQNIKDK~FIHP
NCU03857.1	NN--------------RFRTMASEAFQKIKVKNPVVELDGDE~MTRIIWQDIKEK~FIYP
FG10347.1 	PF--------------AVRTMASST-QKIKVKNPVVELDGDE~MTRIIWQVIKDK~FIHP
AN2999.1  	TARISRPAATRSLYRVQTRTMATEV-QKIKVKNPVVELDGDE0MTRIIWKEIREK0AS--
          	    : .::: :      ****:.. .*************** ******: *::*     

MG07268.1 	YLDIDLKYYDL~GLEYRDKTDDKVTLDAAEAIKKYSVGVKCATITPDEARVKEFNLKQM1
NCU03857.1	FLDIDLKYYDL~GLEYRDQTNDQVTIDAAEAIKKYSVGVKCATITPDESRVEEFKLKQM1
FG10347.1 	YLDIDLKYYDL~GLEYRDETNDQVTIDAAEAIKKYSVGVKCATITPDEARVEEFKLKKM1
AN2999.1  	FLDIDLKYYDL0GLEYRDQTDDKVTTESAEAIKKYGVGVKCATITPDEARVEEFKLKKM1
          	:********** ******:*:*:** ::*******.************:**:**:**:* 

MG07268.1 	WLSPNGTIRNAL~GGTVFREPIVIPRIPRLVPSWQKPIIIGRHAFGDQYRAKDMVVPGEG
NCU03857.1	WLSPNGTIRNAL~GGTVFREPIVIPRVPRLVPGWKKPIIIGRHAFGDQYRAKDLVAPGPG
FG10347.1 	WLSPNGTIRNAL~GGTVFREPIVIPRIPRLVPGWKKPIIIGRHAFGDQYRAKDAVLPGPG
AN2999.1  	WLSPNGTIRNIL2GGTVFREPIVIPRIPRLVPGWTKPIIIGRHAFGDQYRATDRVIPGPG
          	********** * *************:*****.* ****************.* * ** *

MG07268.1 	KLTMTFTPKGGEPQEIQVFDFKNGGGVAQTQYNTDESITGFAQSSFKLALAKGLPLYMST
NCU03857.1	KLQMVYTPEGGEPQVIDVFDYKNGGGVAQTQYNTDESIEGFAHASFKLALDKGLPLYMST
FG10347.1 	KLSMVYTPEGGQPEEIEVFQFKEGGGVAQTQYNTDESITGFAHASFKLAIDKELPLYMST
AN2999.1  	KLELVYTPEGGQPEAIKVFDFP-GGGVTQTQYNTDESIRGFAHASFKLALTKGLPLYMST
          	** :.:**:**:*: *.**::  ****:********** ***::*****: * *******

MG07268.1 	KNAILKKYDGRFKDIFEQLYQSTYKAEFEAKGIWYEHRLIDDMVAQMIKSTGGYIMALKN
NCU03857.1	KNTILKKYDGRFKDIFQQIYDTQYKEAFEAKKIWYEHRLIDDMVAQMIKSSGGYIMALKN
FG10347.1 	KNTILKKYDGRFKDIFQEIYETTYKKEFEAKKIWYEHRLIDDMVAQMIKSSGGYIMALKN
AN2999.1  	KNTILKKYDGRFKDIFQEIFESDYKKEFDAKGIWYEHRLIDDMVAQMIKSEGGFIMALKN
          	**:*************:::::: **  *:** ****************** **:******

MG07268.1 	1YDGDVQSDIVAQGFGSLGLMTSVLITPDGKTFESEAAHGTVTRHYREHQKGRETSTNPI
NCU03857.1	1YDGDVQSDIVAQGFGSLGLMTSVLITPDGKTFESEAAHGTVTRHYREHQKGNQTSTNPI
FG10347.1 	1YDGDVQSDIVAQGFGSLGLMTSVLITPDGKTFESEAAHGTVTRHYREHQKGNETSTNPI
AN2999.1  	1YDGDVQSDIVAQGFGSLGLMTSTLITPDGQAFESEAAHGTVTRHYREHQKGRETSTNPI
          	 **********************.******::********************.:******

MG07268.1 	ASIFAWTRGLIQRGKLDETPDVVAFAETLERACIETVDIDGIMTKDLALACGKTARSDYV
NCU03857.1	ASIFAWTRGLVQRGKLDNTPELIAFAEALEKACIDTVDIDGIMTKDLALACGKTGREDWV
FG10347.1 	ASIFAWTRGLIQRGKLDDTPDVVAFAEGLEQACIDTVDIDGIMTKDLALATGKSERKDYA
AN2999.1  	ASIFAWTRGLIQRGKLDETPDVVKFAEELERACIDVVNEEGIMTKDLALSCGRKERDAWV
          	**********:******:**::: *** **:***:.*: :*********: *:. *. :.

MG07268.1 	TTNEYLDAVERRMKSSLKEKL
NCU03857.1	TTSEYMAAVERRLKSTLKEKL
FG10347.1 	TTNEYLDAVERRLKKTLKEKL
AN2999.1  	TTREYMAAVERRLRANLKARL
          	** **: *****:: .** :*
```
